# Birefringent tissue-mimicking phantom for polarization-sensitive optical coherence tomography imaging

**DOI:** 10.1117/1.JBO.27.7.074711

**Published:** 2022-01-21

**Authors:** Shuang Chang, Jessica Handwerker, Giovanna A. Giannico, Sam S. Chang, Audrey K. Bowden

**Affiliations:** a Vanderbilt University, Vanderbilt Biophotonics Center, Department of Biomedical Engineering, Nashville, Tennessee, United States; b Vanderbilt University Medical Center, Department of Pathology, Microbiology, and Immunology, Nashville, Tennessee, United States; c Vanderbilt University Medical Center, Department of Urology, Nashville, Tennessee, United States; d Vanderbilt University, Department of Electrical and Computer Engineering, Nashville, Tennessee, United States

**Keywords:** phantom, birefringence, optical coherence tomography, polarization, bladder

## Abstract

**Significance:**

Tissue birefringence is an important parameter to consider when designing realistic, tissue-mimicking phantoms. Options for suitable birefringent materials that can be used to accurately represent tissue scattering are limited.

**Aim:**

To introduce a method of fabricating birefringent tissue phantoms with a commonly used material—polydimethylsiloxane (PDMS)—for imaging with polarization-sensitive optical coherence tomography (PS-OCT).

**Approach:**

Stretch-induced birefringence was characterized in PDMS phantoms made with varying curing ratios, and the resulting phantom birefringence values were compared with those of biological tissues.

**Results:**

We showed that, with induced birefringence levels up to $2.1\times 10^{-4}$, PDMS can be used to resemble the birefringence levels in weakly birefringent tissues. We demonstrated the use of PDMS in the development of phantoms to mimic the normal and diseased bladder wall layers, which can be differentiated by their birefringence levels.

**Conclusions:**

PDMS allows accurate control of tissue scattering and thickness, and it exhibits controllable birefringent properties. The use of PDMS as a birefringent phantom material can be extended to other birefringence imaging systems beyond PS-OCT and to mimic other organs.

## 1 Introduction

Appropriate testing platforms are essential to characterize, optimize, and validate new clinical systems and procedures.^1^^,^^2^ The testing of devices and procedures can be performed on a variety of platforms, including calibrated test targets, *ex vivo* human or animal tissues, *in vivo* animal models, tissue-mimicking phantoms, and human subjects. In the early stages of device development, tissue phantoms are often preferred to the alternatives of *ex vivo* tissues and animal models due to their notable advantages of being low cost, easily accessible, non-perishable, free of ethical or legal considerations, and most important, designed with known properties, allowing for repeated and reliable assessment of instruments. As a result, creating realistic tissue substitutes for biomedical applications has been the focus of many ongoing research efforts.

When designing realistic tissue-mimicking phantoms for testing of a device, it is important to include relevant properties of the device to be tested. For example, the acoustic and elastic properties of tissue should be carefully replicated if preparing a phantom for ultrasound imaging^3^; similarly, relevant optical properties of tissue, such as scattering or absorption, should be meaningfully represented if preparing a phantom to be used with light-based imaging modalities.^4^^–^^10^ Notably, pathologies often present as differences in tissue properties. As such, many sophisticated phantoms have been developed to contain structurally dissimilar regions to represent pathologies.^11^^–^^14^

Polarization-sensitive optical coherence tomography (PS-OCT) is a label-free, noninvasive light-based imaging technique that can visualize depth-dependent birefringence properties of tissue.^15^ Recent works have demonstrated that many diseased and normal tissues, particularly epithelial tissues, demonstrate a birefringent contrast that can be detected with polarization-sensitive methods, such as PS-OCT.^16^^–^^18^ For example, several works have shown that the presence of collagen in the bladder wall leads to contrast in the PS-OCT images. This contrast is helpful to differentiate normal from cancerous bladder tissue, since the regularly defined structure of tissue is disrupted by tumor invasion.^18^^–^^20^ However, there have been limited efforts to develop tissue-mimicking phantoms that include realistic, representative birefringent properties. Given the increasing interest and effort being devoted to developing endoscopic systems that are sensitive to polarization, the lack of available, realistic birefringent tissue phantoms poses challenges to conducting controlled and repeated calibration, testing, and validation.

Most existing birefringent phantoms have a major limitation: they are either designed for use with optical systems that lack three-dimensional (3D) imaging capabilities (e.g., forward-scattering systems and Mueller polarimetric systems), or they are fabricated with overly simplified designs lacking meaningful tissue-specific structures.^21^ Birefringent phantoms designed for forward-scattering purposes are too thin and transparent to be compatible with PS-OCT imaging or as representative tissue substitutes.^22^^,^^23^ Phantoms that represent turbid tissue and are used in back-scattering systems, such as Mueller polarimetric imaging, have served as calibrations for multiple polarization parameters. However, they often use quarter wave retarders in place of the birefringent tissue layers, which is not ideal to mimic tissue imaging with PS-OCT given that the retarders have nonbiologically relevant thicknesses and specular reflections that are not characteristic of the backscattering in tissues.^24^

Other phantoms have been designed for use with 3D imaging devices, such as PS-OCT, but have failed to incorporate the biological properties of tissue. These phantoms have been constructed using a variety of techniques (e.g., by stretching and annealing polycarbonate films^25^ or by stretching a rubber phantom^26^^,^^27^); however, they largely exhibited simplified designs: that is, they did not appropriately mimic the layers of the actual biological tissue, nor did they simulate backscattering, which is the basis for tissue imaging in PS-OCT. In fact, the birefringent materials used in these studies exhibited higher backscattering than that of tissue, which compromises the depth of OCT imaging.

In this study, we demonstrate a method for producing tissue-mimicking phantoms for PS-OCT imaging that mimic the birefringent, scattering and thickness properties of layered biological tissues. As an example case study, we designed a flat, multilayered, tissue-mimicking phantom that demonstrates the layered architecture and birefringent properties of the healthy and cancerous bladder tissue in cross section. To this end, we also report the first use of PS-OCT to determine the birefringence of normal and cancerous human bladder tissue and use these values as design criteria in the development of our phantom.

Our new approach to generate birefringent, tissue-mimicking phantoms for PS-OCT will allow for more realistic testing of PS-OCT devices as well as potential opportunities for surgical training. The resulting phantoms are useful in their current form to allow controlled and reliable testing, calibration and comparison of PS-OCT systems with biologically meaningful geometries and light–tissue interaction properties. Moreover, the proposed concept for including birefringence in tissue-mimicking phantoms can be extended to fabricate 3D tissue-mimicking phantoms with realistic organ shapes for the bladder or other organs that contain birefringent structures. While full discussion of a process for fabrication of a 3D birefringent phantom is beyond the scope of this paper, we provide some inspiration for relevant methods to create such phantoms as future directions.^28^^,^^29^

## 2 Birefringence in Human Bladder Tissue

**Fig. 1:**
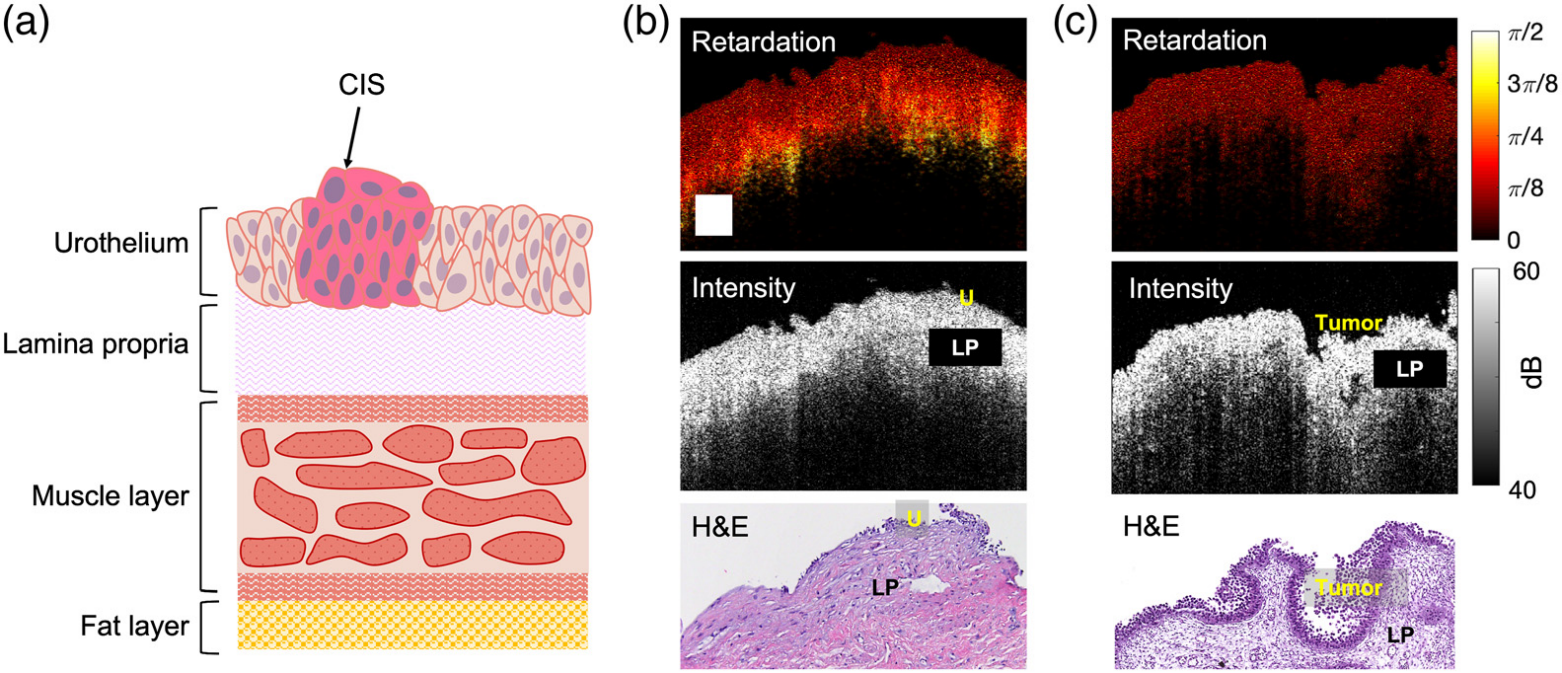
(a) Diagram showing layers of the bladder. (Note that the diagram was not drawn to scale.) Example retardation mapping of (b) normal and (c) diseased bladder sample, compared with conventional OCT intensity images and H&E histology. Scale bar=0.2  mm.

A healthy bladder wall contains several layers of varying thickness. The top layer, called the urothelium, is five to seven cell layers thick and appears in PS-OCT as a thin, dim layer ∼50  μm in thickness. Just below the urothelium is the lamina propria (LP) layer. The LP of human urinary bladder contains collagen fibers and is birefringent in healthy individuals. It is typically 300- to 400-μm thick. Finally, the muscularis propria (MP) layer, the third visible layer in OCT images, is 1.6-mm thick; PS-OCT systems cannot generally image to the bottom of the MP due to light attenuation and a limited imaging depth.^29^ Additionally, the bladder has a perivesical fat layer that surrounds the outside of the bladder. An example diagram of the layers of the bladder wall is shown in Fig. 1(a).

To determine the birefringence of bladder tissue, we conducted a study approved by the Institutional Review Board of Vanderbilt University (IRB# 191337) and obtained informed consent from all study subjects. We enrolled patients presenting to the Vanderbilt University Medical Center with confirmed bladder cancer or suspicious bladder lesions that were scheduled to receive a transurethral resection of bladder tumor procedure. One healthy tissue sample and one pathological [atypical or carcinoma *in situ* (CIS)] tissue sample were collected from each enrolled patient.

All tissues were immersed in saline upon resection and during imaging. Fresh tissue samples (within 30 min of resection), with sizes ranging from ∼4 to 6 mm in diameter, were imaged with a commercial PS-OCT system (TEL220PSC2, ThorImage OCT software version 5.3.2.0, Thorlabs, Inc.) using an OCT-LK3 objective at an A-scan rate of 28 kHz. The incident light on the sample was circularly polarized with a center wavelength of 1300 nm. The measured axial and lateral resolution of the system were 5.5 and 7.8  μm, respectively. An immersion type Z-spacer (OCT-IMM3, Thorlabs, Inc.) was attached to the objective to allow imaging at a close distance to the tissue surface while reducing the strong back reflections. The output of the system includes both conventional, intensity-based OCT images, as well as images of the retardation and optic axis mappings calculated from the image data, which characterize the birefringence properties of the sample. Immediately after imaging, the tissues were fixed and processed for histology. Hematoxylin and eosin (H&E) staining was used for tissue type confirmation.

We could compute the sample birefringence Δn from the linear relationship between retardation versus depth in the following equation:

δ=2πΔnz/λ, (1)

where λ is the center wavelength of the incident light, z is the distance of light traveled in the material, and δ is the cumulative phase retardation. To extract the birefringence from each layer of the bladder samples, the tissue surface in the PS-OCT image was first determined by intensity thresholding with Otsu’s method in MATLAB (MathWorks, Inc.). For normal tissue, this surface corresponded to the urothelium layer. We then relied on a simple segmentation for identifying the location of the LP layer in the tissue, where we assumed the urothelium layer extends 50  μm below the surface and the LP region extends 200  μm below the urothelium. After aligning the surface position across different lateral locations, we averaged the retardation laterally over a 2-mm region of the B-scan, and the birefringence was extracted from the slope of a line fitting the retardation versus depth.

The mean and standard deviation of the birefringence values measured from the urothelium (n=11 and mean age=69.6) and LP layer of normal (n=12 and mean age=69.8) human *ex vivo* bladder biopsies were found to be $2.54\times 10^{-5}\pm 8.88\times 10^{-5}$ and $1.18\times 10^{-4}\pm 5.43\times 10^{-5}$, respectively. Note that some birefringence measurements in the urothelium were “negative” (a negative fitting slope), likely due to the facts that (1) the layer is very thin, and (2) the birefringence is very close to zero. These facts also explain the relatively large standard deviation for the urothelium measurements and suggest that the urothelium layer is not birefringent. The mean and standard deviation of the birefringence values measured from the LP layer in the diseased tissues (CIS tumors, n=6, and mean age=77.3) was $3.21\times 10^{-5}\pm 2.27\times 10^{-5}$. As this value also suggests very minimal to negligible changes of retardation versus depth, we considered the LP layer to be nonbirefringent for the purpose of phantom design. Due to the strong signal attenuation near the MP, the birefringent property of the MP was not analyzed with PS-OCT. However, because of the presence of smooth muscle fibers in the MP and since CIS is not muscle invasive, the MP layer of the bladder should exhibit birefringence and is not affected by the disease condition. Example OCT intensity and retardation maps of normal and diseased bladder tissues are shown in Figs. 1(b) and 1(c), respectively.

## 3 Birefringence in a Tissue-Mimicking Material

### 3.1 Choice of Material

Optical properties (e.g., reflectivity, scattering, and absorption) reveal heterogeneities in tissue and allow separation of dissimilar tissue types. A number of previous research efforts have focused on providing mathematical and experimental details for calibration and fabrication of the scattering and absorption properties in phantoms,^5^^,^^30^^,^^31^ mainly by means of mixing an appropriate weight fraction of scattering agents [e.g., titanium dioxide (TiO_2_)] and absorption agents (e.g., India ink).^32^ A common material for tissue-mimicking phantoms is polydimethylsiloxane (PDMS) due to its tunability for scattering and absorption.^33^^,^^34^ PDMS is particularly useful for phantoms that mimic highly stretchable organs, such as the bladder.^29^

**Fig. 2:**
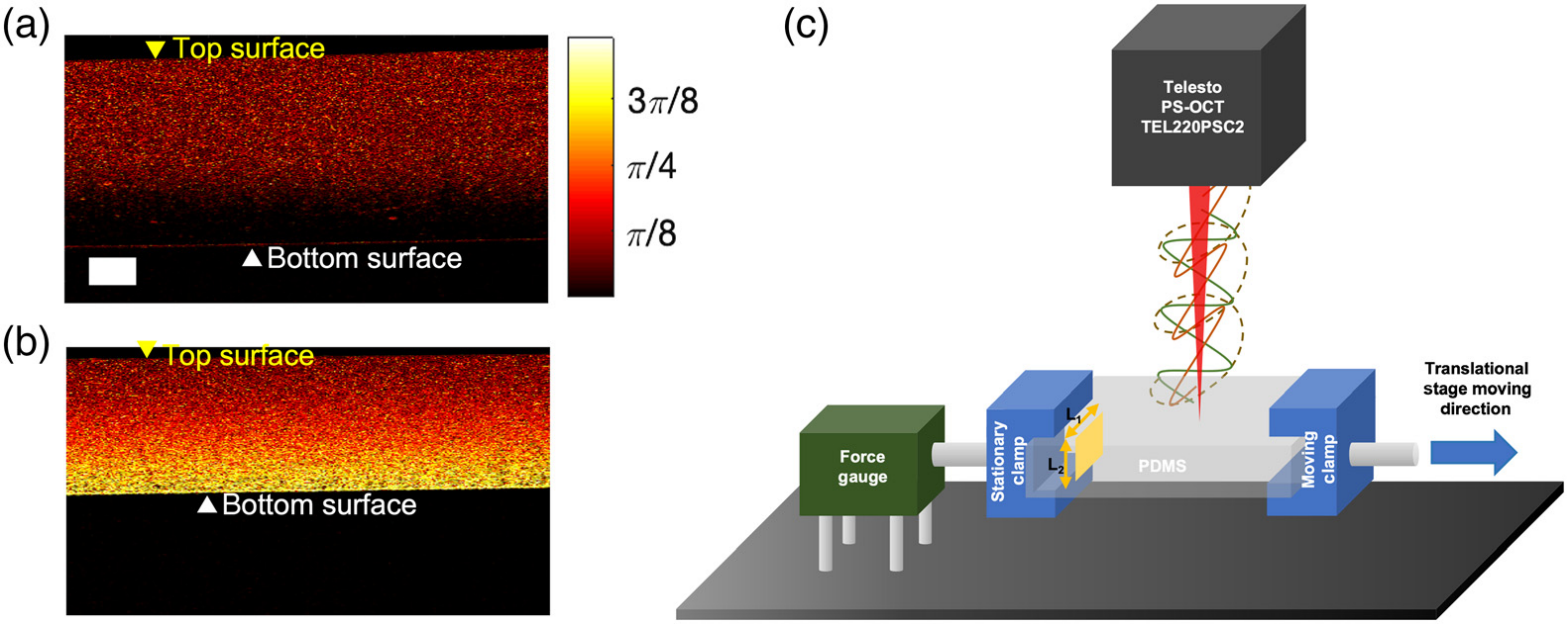
Retardation maps of PDMS in relaxed (a) and stretched (b) states. Retardation plots show the cross-sectional plane of the PDMS. Yellow and white triangles point to the location of the top and bottom surface of the phantom, respectively. Scale bar=0.2  mm. (c) Imaging system setup for measuring phantom birefringence.

Our strategy to introduce birefringence in a tissue-mimicking phantom relies on the photoelastic property of PDMS, which varies as a function of the curing ratio and stretch. Photoelasticity describes the induced macroscopic birefringence in a material undergoing a large elastic deformation.^35^ When subject to a mechanical force, the polymer chains in soft materials, such as hydrogels and elastomers, increase in alignment and exhibit optical anisotropy, which causes a difference between the refractive indices (Δn, or birefringence) for different polarization states. The high elasticity of PDMS allows for large deformation with minimal stress relaxation. As a result, PDMS-based structures can be repeatedly stretched without losing their induced birefringence over time.^36^ Figure 2 shows representative PS-OCT-generated retardation maps of single layer, homogeneous PDMS phantoms in relaxed and stretched states. The phantoms shown in Figs. 2(a) and 2(b) were made with a 15:1 weight ratio of base to curing agent, comprised 0.2 weight ratio (w%) ${\mathrm{TiO}}_{2}$ and had an initial (relaxed or unstretched) thickness of 1.5 mm.

### 3.2 Design and Preparation of Birefringent Slab Phantoms

To characterize the stretch-induced birefringence of PDMS phantoms, we first prepared several single layer, scattering phantoms (i.e., slab phantoms) from Sylgard 184 silicone elastomer (Dow Silicones Corporation) by mixing the base solution (part A) and the curing agent (part B) at specific curing ratios. In this study, we prepared PDMS phantoms with four curing ratios (weight ratios of part A to part B): 10:1, 15:1, 20:1, and 25:1. We also added ${\mathrm{TiO}}_{2}$ to mimic tissue scattering. The weight ratios used included 0.04, 0.15, 0.2, and 0.3 w% and were chosen based on values of the light attenuation coefficient (AC) determined in previous literatures for the different layers in bladder tissues.^28^ Each solution was degassed in a desiccator before being transferred to a plastic mold to cure at 80°C for 2.5 h.

### 3.3 General Procedure for Measuring Birefringence in Slab Phantoms

**Fig. 3:**
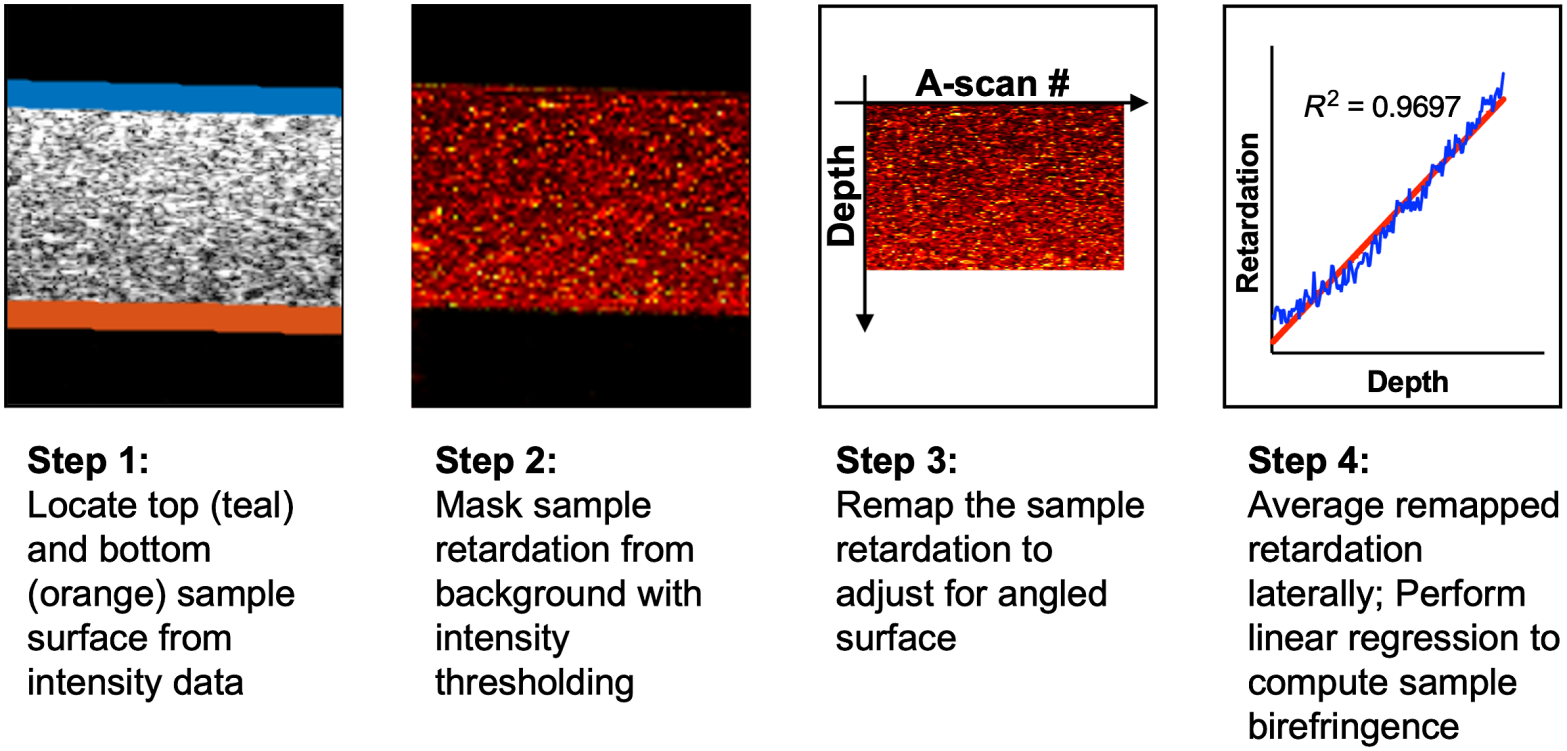
Flow diagram for calculation of phantom birefringence.

We then used the testing setup shown in Fig. 2(c) to measure the retardation as a function of applied stretch. The PDMS phantom was secured into two clamps with an initial clamp separation of 6 mm, which we refer to as the “original length.” One clamp was held stationary by mounting it to a force gauge (FG-3007, Nidec-Shimpo, Inc.); the other clamp was mounted onto a translational stage and allowed to move away in 2.5-mm increments using the micrometer, which controlled the amount of stretch. The imaging location was centered on the phantom and remained unchanged for a given stretching experiment. The phantom was imaged when relaxed and then at each stretch increment. From the PS-OCT image, we determined the thickness and birefringence for each phantom and experimental condition in the following manner (Fig. 3):

1. The top and bottom surfaces of the phantom were automatically segmented by intensity thresholding. In cases where the bottom surface was not visible on the B-scan, a maximum detection depth was determined through intensity thresholding and was used in the birefringence determination. We used the number of pixels between the top and bottom layer as a relative thickness measurement; this was converted to a physical value by assuming a refractive index to be 1.4. Although the refractive index is expected to vary slightly for phantoms with different curing ratios, our birefringence measurement is based on the relative change in thickness, so the exact coefficient for the pixel-to-thickness conversion is irrelevant.
2. We used the surface pixels to create a mask for the retardation map generated by PS-OCT.
3. We aligned the surface from each lateral position so that the phantom was always perfectly horizontal in the corrected image.
4. We averaged the retardation laterally at each depth from the surface and used linear regression to determine the slope of the retardation versus depth, from which we derived the birefringence. For a given amount of stretch, the measured birefringence and thickness of the PDMS phantoms were found to be roughly uniform in the area of interest. Therefore, in our characterization experiments, we assumed homogenous birefringence in the area of interest on the phantom at a given amount of stretch.

We further determined the stress σ applied for each amount of stretch from force measurements recorded by the force gauge using the following equation:

σ=P/A, (2)

where P is the stretching force and A is the cross-sectional area of the phantom in its relaxed state. The cross-sectional area was taken to be the initial phantom thickness ($L_{2,0}$) multiplied by the width of the phantom ($L_{1}$), which is width of the clamp that was in contact with the phantom, where $A=L_{1}\times L_{2,0}$. Note, we assumed negligible changes in the width measurement throughout the experiment.

### 3.4 Characterization of Slab Phantom Birefringence

The optical anisotropy of PDMS is determined by the number of cross-linked polymer chains in a unit area, which can be adjusted by varying the weight ratio of the base solution (part A) and the curing agent (part B). Theoretical prediction and experimental data of birefringence induced in PDMS have been studied recently,^35^ albeit with very modest amount of stretch. As a result, the published birefringence levels are too low to be useful in mimicking values of tissue birefringence. In this study, we explored a wider range of stretch values in slab PDMS phantoms of varying curing ratios and measured the induced birefringence under each condition.

**Fig. 4:**
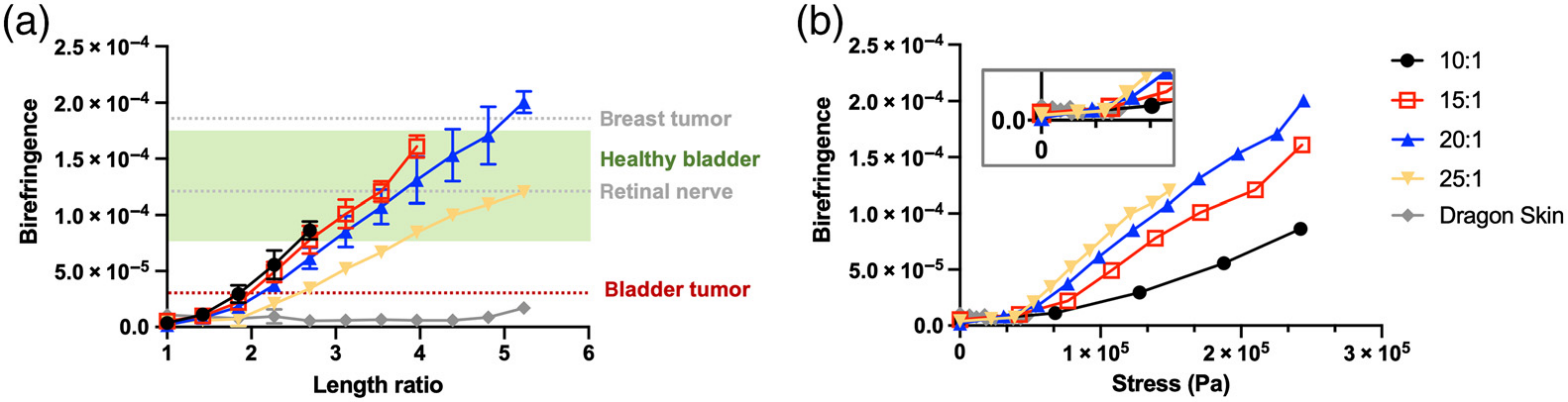
(a) Birefringence versus stretch and (b) birefringence versus stress relationships at different curing ratios, characterized with 1.5-mm-thick phantoms with 0.2 w% ${\mathrm{TiO}}_{2}$.

To characterize the amount of stretch, we defined a parameter, the length ratio, which equals the elongated length divided by the original length. For each curing ratio, the induced birefringence versus stretch relationship was measured and plotted as a function of length ratio, as shown in Fig. 4(a) for phantoms with an initial thickness of 1.5 mm. Each data point represents the average birefringence and standard deviation measurements from five measurement repeats. Note that error bars are not shown for data points with very low standard deviations, as in these cases the error bars are smaller than the extent of the data point. The results reveal that PDMS phantoms made with lower curing ratios (i.e., 10:1) have greater stiffness and are therefore more resistant to deformation. The maximum achievable elongation, that is, the point at which the phantom starts to slip out of the clamp, is lowest for 10:1 phantoms, followed by that for 15:1; meanwhile, 20:1 and 25:1 phantoms exhibit greater stretchability and can reach more than five times their original length. We reason that both the increase in stiffness as well as the decrease in stickiness contribute to the slipping that happens at lower curing ratios, such as the 10:1.

In our experiments, all four curing ratios exhibited birefringence changes with stretch. At lower curing ratios, the induced birefringence increases at a faster rate with stretch than at higher curing ratios. However, because of the low stretchability of low curing-ratio phantoms, higher values of birefringence could ultimately be obtained in stretched phantoms made with higher curing ratios. For example, the largest birefringence we induced ($2.1\times 10^{-4}$) over the range of length ratios we explored was achieved with 20:1 phantoms, when stretched to approximately five times the original length. Notably, birefringence levels we induced in PDMS are within the range of those in tissues that exhibit weak birefringence, including the normal and diseased bladder (see Sec. 2), retinal nerve ($\Delta n=1.2\times 10^{-4}$),^37^ and tumor in breast tissue ($\Delta n=1.8\times 10^{-4}$).^38^ We also report the birefringence properties of another silicone-based polymer, Dragon Skin (Smooth-On, Inc.). Under the same stretching conditions, the 1.5-mm-thick Dragon Skin phantom showed negligible changes in birefringence, and we therefore concluded that the Dragon Skin does not exhibit a stretch-induced birefringent property.

We also measured the force exerted on the PDMS phantom as we performed the stretching experiments and determined the changes of birefringence as a function of stress, as shown in Fig. 4(b). The stress measurement is determined by both the force and the original cross-sectional area, which is related to the original phantom thickness, ($L_{2,0}$). Stress measurements on the Dragon Skin were also performed; however, the elasticity of Dragon Skin is much lower than PDMS, and thus exhibited negligible birefringence changes over the applicable range of stress.

**Fig. 5:**
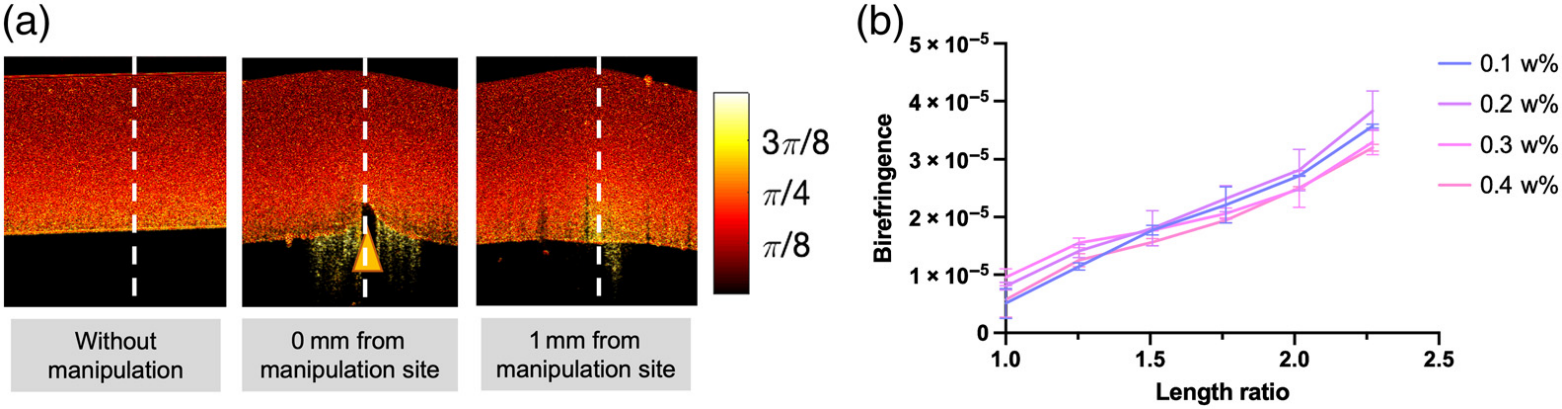
(a) Retardation maps of cross section of PDMS when no manipulation is applied (left image), and when there is manipulation applied: at an imaging plane that includes the tweezer (0 mm from manipulation site, middle image) and a parallel imaging plane that is 1-mm away from the tweeze (right image). The orange triangle indicates the location of the tweezer, which simulates manipulation with surgical tool. The white dashed lines indicate location where birefringence measurements were taken. (b) Birefringence versus stretch at varying ${\mathrm{TiO}}_{2}$ (w%), characterized with 1.5-mm-thick phantoms made with 20:1 curing ratio.

Tissue-mimicking phantoms are particularly useful for testing tools and procedures in a mock surgical environment. As manipulation of tissue with surgical tools can cause local changes in the measured birefringence at the manipulation site, we also tested whether the use of PDMS to mimic tissue birefringence allows resemblance of such changes. We used a tweezer to press perpendicularly on a layer of stretched PDMS (15:1 curing ratio, 1.5-mm thick) and, without piercing the phantom, we imaged it from the opposite side of the tweezer at two cross-sectional imaging planes: (1) the plane, including the tweezer, i.e., the manipulation site, and (2) a parallel imaging plane located 1-mm away from the tweezer. We pressed from the opposite side of the imaging surface to allow better visualization of the effect on measured retardation (if imaged from the same side, the tweezer would cast a shadow and prevent changes directly underneath the tweezer to be visualized). The 1-mm-away imaging plane was used to study the whether the effect of manipulation can change the birefringence measurements from the surrounding regions. As shown in Fig. 5(a), a local birefringence change can be observed as a change in the retardation mapping, when comparing the cross-sectional images resulted from no manipulation and when manipulation was applied. In this case, the retardation increases more rapidly along the axis of manipulation than the surrounding PDMS and untouched PDMS. To quantify the amount of change in birefringence, we took birefringence measurements from 20 A-scans centered at the manipulation site and 20 A-scans from the corresponding lateral location of the untouched PDMS. The locations of the 20 A-scans are indicated by the white dashed line in Fig. 5(a). We observed a 38.6% and 35.6% increase at a 0- ($\Delta n=1.40\times 10^{-4}$) and 1-mm distance ($\Delta n=1.37\times 10^{-4}$) from the manipulation site, respectively, when compared with untouched PDMS ($\Delta n=1.01\times 10^{-4}$).

A major advantage of using PDMS as the birefringent material is that it is optically clear, which grants easy control of the scattering properties. By means of tuning the volume of scattering in each representative layer, we can achieve realistic tissue contrast in conventional B-scan intensity images. To confirm that the induced birefringence measured with PS-OCT is not dependent on the amount of scatterers, we conducted stretching experiments with slab phantoms comprising 0.1 w% to 0.5 w% ${\mathrm{TiO}}_{2}$, while keeping all other factors constant (i.e., curing ratio and thickness). The results suggest that there is no significant difference in the rate of birefringence increase with scatterer weight ratio, as shown in Fig. 5(b). This result confirms that we can tune the scattering of the phantom, as is necessary to mimic different types of biological tissue, without altering the birefringence levels.

## 4 Design of a Planar Bladder Tissue-Mimicking Phantom

### 4.1 Relevant Properties of the Human Bladder

**Fig. 6:**
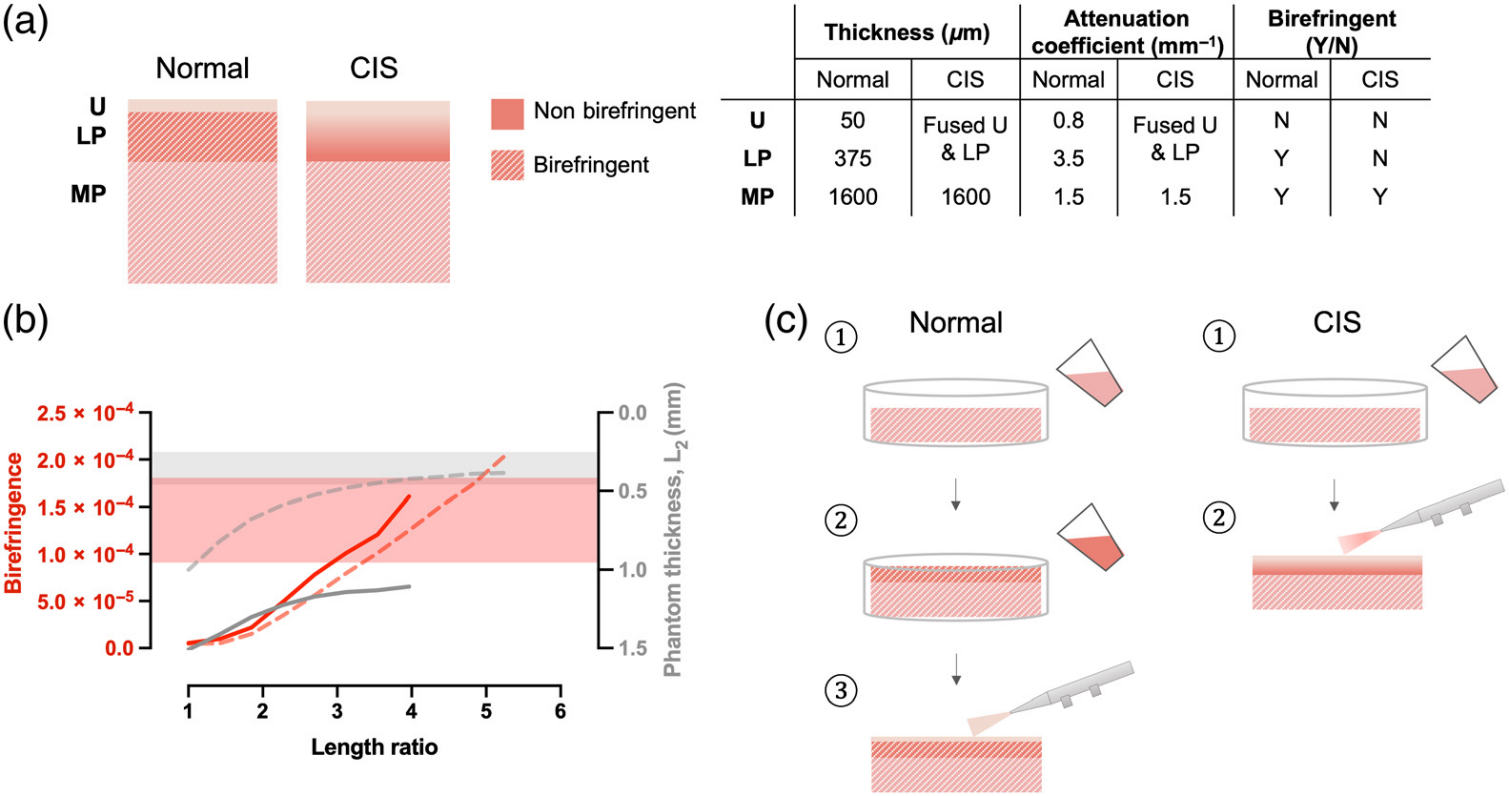
(a) Overall design of desired thickness, AC, and birefringence in each layer of the bladder under normal and tumor conditions. (b) Induced birefringence in 15:1 phantoms with different initial thicknesses (solid line: 1.5-mm-thick phantom and dashed line: 1-mm-thick phantom). Pink shaded region: birefringence values observed in normal bladder LP. Gray shaded region: thickness values observed in normal bladder LP. Overlap region shows birefringence and thickness values to be achieved in the LP layer of normal bladder phantoms. (c) Fabrication steps for normal (left) and diseased (right) bladder phantoms.

To mimic the layered structure of the bladder wall under PS-OCT imaging, there are three important design criteria for each layer: thickness, AC, and level of birefringence. When imaged from the lumen side, the three layers in the healthy bladder can be distinctively visualized and have thicknesses of 50  μm, 400  μm, 1.6 mm, and ACs of 0.8, 3.5, and $1.5\;{\mathrm{mm}}^{-1}$, respectively.^28^^,^^29^ Since the urothelium is a cell layer, it does not exhibit any birefringence, which is consistent with our finding of negligible birefringence in Section 2. In contrast, the collagen-rich LP layer and the smooth muscle in the MP layer both exhibit birefringence in healthy bladders. In early stage cancerous development, bladder tumors, such as in the case of CIS, have reduced contrast between the urothelium and LP on the OCT image, which causes them to lose their distinct stratification and appear as a fused layer. Dimming in the OCT intensity and near complete loss of birefringence can also be observed as the result of cancer development,^39^ which is consistent with our finding of negligible birefringence in the fused CIS layer of the diseased bladder in Sec. 2. The table in Fig. 6(a) summarizes these general characteristics and inspires our phantom design.

To generate layers that do not exhibit birefringence with stretch, we chose to use the Dragon Skin as the material for nonbirefringent regions, since it does not exhibit photoelasticity but is still highly elastic. Hence, Dragon Skin was used for the urothelium layer of the healthy bladder phantom. Similarly, we chose to use multiple layers of Dragon Skin with ACs ranging from 1.8 to $2.2\;{\mathrm{mm}}^{-1}$ (lower AC near the MP and higher AC at the top) to represent the fused appearance of the urothelium and LP layers in the CIS-mimicking phantom, given that these layers also exhibit negligible birefringence in the diseased case. The varying AC levels were achieved by adding different weight ratios of ${\mathrm{TiO}}_{2}$, of which the relationship has been previously established.^28^

Because of the presence of collagen and smooth muscle fibers, respectively, both the LP and MP layers of the healthy bladder exhibit birefringence. Hence, both layers should be fabricated from PDMS. To determine the curing ratios to use for these layers, we consider the graph in Fig. 6(b), where we compared the birefringence of stretched phantoms with initial thicknesses of 1 (dashed line) and 1.5 mm (solid line) and reported the change of thickness of the phantom ($L_{2}$) as a function of length ratio. The red shaded region denotes the range of birefringence values associated with a healthy bladder LP, while the gray shaded region denotes the thickness value of a typical LP layer. We chose to use a curing ratio of 15:1 for the LP layer, as it is capable of achieving birefringence values that fall within the range for healthy LP with modest length ratios. Although we could not measure the birefringence of the MP layer directly with PS-OCT due to the high attenuation of light when reaching that depth, we chose to use a curing ratio of 25:1 to reflect the non-negligible, but lower expected birefringence in that layer in healthy tissue. Because CIS is a superficial tumor that does not extend to the MP, we chose to use the same recipe for the MP of the diseased phantom as for the healthy phantom.

### 4.2 Phantom Fabrication Procedure

Figure 6(c) shows the steps taken to fabricate the healthy and CIS phantoms. First, the MP layers of both phantoms were fabricated by pouring ${\mathrm{TiO}}_{2}$-infused PDMS (0.15 w%) into a standard mold (e.g., a petri dish) and letting it cure. Note that we did not tightly control the thickness of this layer because it is typically too thick to visualize in OCT, so its actual thickness does not matter so long as it exceeds 1.6 mm when stretched. Our choice of a high curing ratio for this layer (25:1) ensures that it can stretch well. For the healthy phantom, the LP layer was added and cured atop the MP by pouring another layer of PDMS with ${\mathrm{TiO}}_{2}$ (0.3 w%). Its thickness was controlled by weight versus volume calibration. Finally, to create a thin urothelium layer, a ${\mathrm{TiO}}_{2}$-inflused Dragon Skin solution (0.04 w%) was first thinned with NOVOCS solution (Smooth-On, Inc.) at 100 w% (i.e. part A + part B : NOVOCS = 1:1) and then sprayed onto the LP layer with a Badger air brush (200-BWH) and Badger air compressor (AS180-12) at the rate of one second per spray coating, until the thickness reached the design criterion for the urothelium (∼90  μm).^28^ To fabricate the combined urothelium and LP layers in the disease phantom, the same air brushing technique was used. In this case, multiple coatings of Dragon Skin were applied with ACs ranging from 0.15 w% to 0.2 w%: the AC of the solutions decreased progressively from near the MP toward the tissue surface. Sonication was used during Dragon Skin preparation to ensure evenly dispersed ${\mathrm{TiO}}_{2}$ particles. All Dragon Skin layers were allowed to cure at room temperature for 6 h.

### 4.3 Measurements in the Final Phantoms

**Fig. 7:**
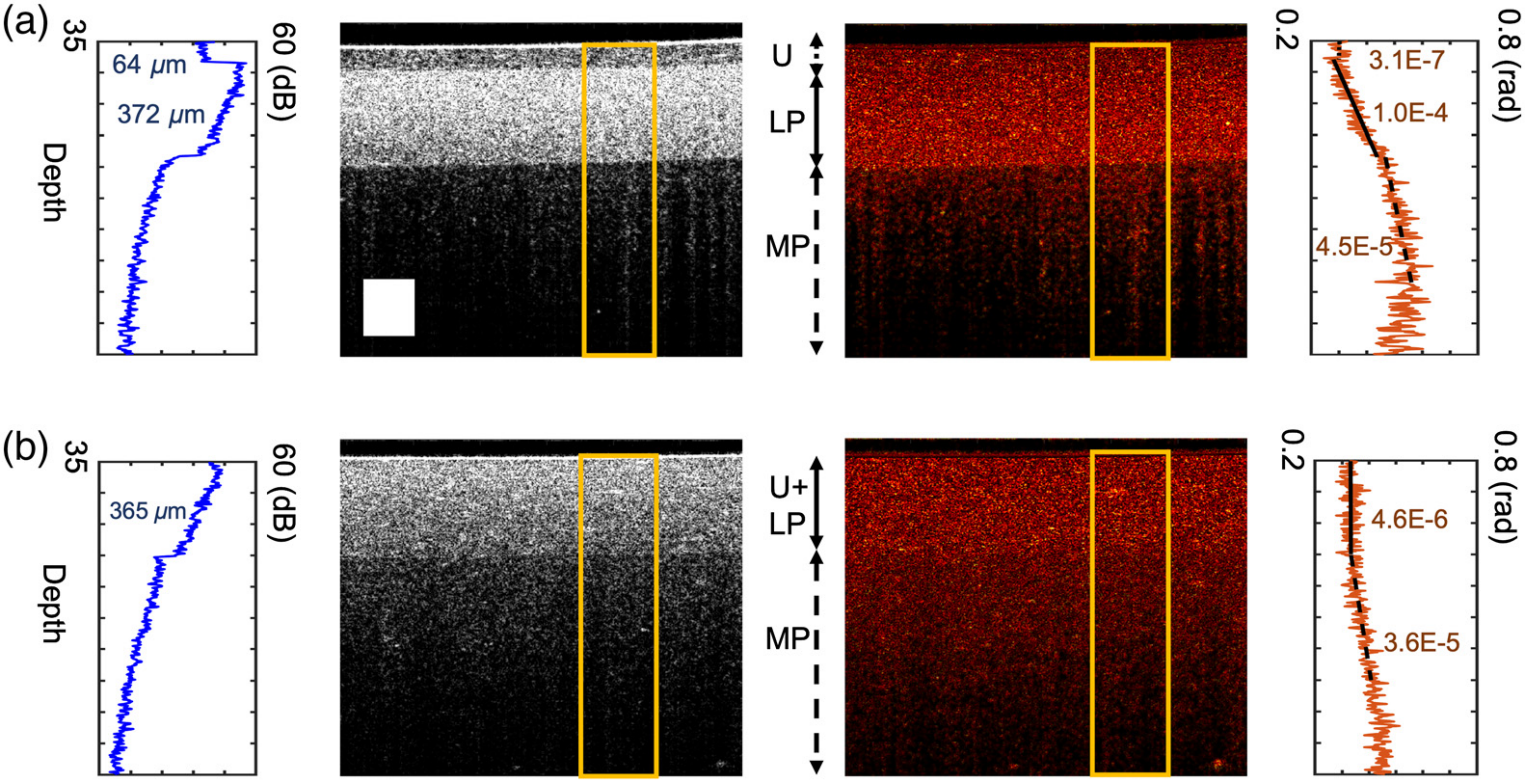
B-scan and retardation maps of (a) normal and (b) diseased bladder phantoms. Plots of averaged intensity and retardation versus depth plots are shown for the regions boxed with yellow solid line. Scale bar = 0.2 mm.

OCT intensity and PS-OCT retardation images of the final normal and diseased phantoms are shown in Fig. 7. In this image, both phantoms were stretched to a length ratio of nearly four. Intensity and retardation versus depth plots, averaged laterally in the boxed regions (and calculated from the surface of the phantom to 500 pixels below the surface), are shown next to the OCT intensity cross-sectional images on the left. Both the intensity and the retardation map can visually differentiate the normal from the diseased condition, suggesting that the design criteria have been met successfully. The achieved thicknesses of the layers and measured birefringence are reported on the plot. Specifically, in the normal bladder phantom, we achieved birefringence levels of $3.11\times 10^{-7}$, $1.05\times 10^{-4}$, and $4.50\times 10^{-5}$ in the urothelium, LP, and MP layers. In the diseased phantom, the values for the fused layer and MP are $4.61\times 10^{-6}$ and $3.59\times 10^{-5}$.

## 5 Discussion

Although not shown in this paper, the fabrication process we introduced can be easily expanded to show other pathological conditions in the bladder, such as inflammation (characterized by a thickened urothelium and reduced birefringence in the LP) and T2 (muscle-invasive) stage tumors (complete loss of stratification and birefringence in the top three layers of the bladder).^28^ Other works have already described methods to mimic the appearance of bladder rugae, such as through use of crumpled aluminum foil during the molding process, and to improve the visual appearance of PDMS and Dragon Skin solutions to better resemblance actual tissue under white light illumination.^28^^,^^29^ These strategies can be equally applied to create a more realistic bladder phantom. In the future, we will consider developing a fully 3D bladder phantom to aid the testing of endoscopic PS-OCT devices. In this case, stretching (necessary to induce birefringence) can be accomplished by filling the phantom with water or saline, which also serves to mimic the realistic clinical environment, as patient bladders are usually distended with saline prior to cystoscopy examinations.

While the proposed strategy is useful to mimic birefringence levels associated with weakly birefringent tissues, the range of parameters we explored is not sufficient to mimic tissues with more orderly aligned structures, such as human skin ($\Delta n=1.2\times 10^{-3}$), scar tissue ($\Delta n=0.9\times 10^{-3}$),^40^ and myocardial fiber ($\Delta n=0.69\times 10^{-3}$ to $1.46\times 10^{-3}$).^41^ Achieving greater levels of birefringence with PDMS may require new fabrication strategies, such as adding nanofibers to induce greater birefringence with deformation,^42^ which may allow the resulting PDMS to reach the birefringence levels needed to mimic highly birefringent tissues. Alternatively, one may consider the use of other materials that have been described in previous literatures to induce high birefringence.^25^

## 6 Conclusions

In this study, we describe a method for inducing birefringence in PS-OCT phantoms with a common material used in phantom development—PDMS. We characterized the level of induced birefringence in PDMS slab phantoms with stretch and showed that the values could reach the range of many weakly birefringent tissues. The major advantage of using PDMS as the birefringent material is that it permits precise control of the scattering properties and phantom thickness, which allows fabrication of sophisticated phantoms that realistically mimics tissue structures and light–tissue interactions. We demonstrated only layered slab phantoms in this work. However, 3D birefringent phantoms that resemble shapes of hollow organ, such as the bladder, can also be achieved with the proposed method. Air or water inflation may be used to cause deformation in 3D PDMS phantom, which removes the need of using clamps. In summary, given the wide availability and the extensive use of PDMS in phantom development research, this method can be readily adopted in other birefringent tissue phantom designs and used for polarization imaging systems beyond PS-OCT.
